# Rhabdomyolysis Causing Renal Failure Following Cardiopulmonary Resuscitation, Cardioversion, and Myocardial Infarction: A Case Report and Review of the Literature

**DOI:** 10.7759/cureus.12666

**Published:** 2021-01-12

**Authors:** Sonia Gupta, Vinay Kumar Thallapally, Joseph Thirumalareddy

**Affiliations:** 1 Internal Medicine, Creighton University, Omaha, USA; 2 Internal Medicine, Creighton University School of Medicine, St. Joseph's Hospital and Medical Center, Omaha, USA

**Keywords:** cardiopulmonary resuscitation, cardioversion, myocardial infarction, rhabdomyolysis, acute renal failure

## Abstract

Rhabdomyolysis is a condition where there is damage of skeletal muscle, causing myoglobin leak into the circulation. We report a case of a 69-year-old female with a history of hypertension, hyperlipidemia, diabetes mellitus, morbid obesity, paroxysmal atrial fibrillation, and chronic kidney disease stage who underwent cardiopulmonary resuscitation following ventricular fibrillation to restore effective cardiac rhythm. After the third attempt of defibrillation she converted to sinus rhythm. Her echocardiography was suggestive of myocardial infarction (MI). On the second day of her hospitalization, she started becoming oliguric and her creatinine started rising up causing acute kidney injury (AKI). The patient’s creatinine kinase (CK) level peaked at 6380 u/L (normal range 26-192 u/L), myoglobin was >20,000 ng/mL (normal range 9-83 ng/mL), and myocardial bound (MB) isoenzyme of CK was 4.5 ng/mL (normal range 0-3.6 ng/mL). Plasma creatinine increased to 5.71 mg/dL and ultimately developed renal failure. She was started on hemodialysis. Her cardiac catheterization was suggestive of MI.

Our case highlights that MI, cardiopulmonary resuscitation, and cardioversion can be a cause for myoglobinuric renal failure, which has been rarely reported in the literature before.

## Introduction

Rhabdomyolysis is a condition where there is damage of skeletal muscle, leading to leakage of toxic cellular contents into the circulation. It is characterized by marked elevation of creatine kinase (CK) five to ten times above the normal limit of normal serum levels [1]. Both traumatic and nontraumatic etiologies have been recognized [2]. Acute renal failure and rhabdomyolysis has been well recognized [3]. Myocardial infarction (MI), cardio-pulmonary resuscitation (CPR), and cardioversion have been reported to cause myoglobinuria but very few case reports [4-5] have been reported to cause myoglobinuric renal failure. This case reports the course of a patient leading to renal failure after CPR and cardioversion due to MI.

## Case presentation

A 69-year-old female with a past medical history of hypertension, hyperlipidemia, type 2 diabetes mellitus, morbid obesity, and chronic kidney disease stage 3A with baseline creatinine of 1.3-1.9 was complaining of shortness of breath for two days and was found unresponsive at home by her husband. Emergency medical services were called. En-route to the emergency room, she was found pulseless and initial rhythm showed ventricular fibrillation. The patient received CPR for 45 minutes, direct current cardioversion counter shocks of 360 J each, total of 1080 J were implemented and multiple boluses of epinephrine, amiodarone, lidocaine, and magnesium were given to restore effective cardiac rhythm. After the third attempt of defibrillation she converted to sinus rhythm. She was then transferred to the ICU. Her initial arterial blood gas was suggestive of respiratory acidosis with pH of 7.14, Pco2 of 84, and Po2 of 98. She was intubated with pressure support mechanical ventilation with the following settings: FiO2 of 100 %, respiratory rate of 32, tidal volume of 400 mL, and positive end expiratory pressure of 5. Her vital signs after the event were as follows; blood pressure of 110/80 mmHg, pulse was 110/min, and oxygen saturation was at 95% with the ventilation setting. Electrocardiogram after the event showed sinus tachycardia. Chest roentgenogram was suggestive of mild pulmonary edema. Her troponin level was 0.18 ng/mL (normal range <0.04 ng/mL) followed by 0.44 ng/mL and 0.43 ng/mL. On examination the patient was minimally following command. Her breath sound was coarse bilaterally with no other remarkable physical examination findings. Her initial laboratory work up showed potassium of 4 mmol/L (normal reference 3.7-5.1 mmol/L), blood urea nitrogen of 35 mg/dL (normal reference 6-24 mg/dL), and creatinine of 1.70 mg/dL (normal reference 0.50-1.10 mg/dL). Echocardiography of the heart showed ejection fraction of 50%-55% and motion wall abnormality of basal, mid anterior, and inferior wall suggestive of underlying MI, which was a new finding as compared to the old report. Cardiac catheterization could not be performed due to her critical condition. On second day of her hospitalization, she started became anuric (urine output <100ml/24 h) and her creatinine started rising up causing acute kidney injury (AKI). Her further laboratory works up during the hospital course are listed in the table (Table 1). Her urinalysis showed no red blood cells, pyuria or bacteria, but did show some granular casts. A renal ultrasound was negative for obstruction, and her fractional sodium was found to be 4.5%. The patient’s CK level peaked at 6380 U/L (normal range 26-192 U/L), myoglobin was >20,000 ng/mL (normal range 9-83 ng/mL), and myocardial bound (CK-MB) isoenzyme of CK was 4.5 ng/mL (normal range 0-3.6 ng/mL). The patient’s renal function did not improve with fluid loading as a part of resuscitation post CPR initially considering it to be prerenal cause or with stress dose furosemide (1 mg/kg) after the diagnosis of intrinsic AKI was made. However, her plasma creatinine rose to 5.71 mg/dL. She was started on hemodialysis. However, her kidneys continued to show minimal renal recovery and the patient was transferred to a long-term care facility. Elective left heart catheterization was done after a month later as per cardiology recommendation. She was found to have total occlusion in the right coronary artery (RCA) and received percutaneous coronary intervention to RCA. However, her renal recovery has been minimal and the patient still receiving hemodialysis three times a week.

**Table 1:** Laboratory markers of the patient since admission. BUN, blood urea nitrogen; CK, creatinine kinase; LDH, lactate dehydrogenase; AST, aspartate aminotransferase; ALT, alanine transaminase

Normal range	11/20 (day of admission)	11/21	11/22	11/24	11/25	11/27	11/30	12/2	12/5
Sodium (135-145 mmol/L)	138	139	140	138	139	138	140	137	139
Potassium (3.7-5.1 mmol/L	4	5.3	4.7	5	5.1	3.8	3.8	3.9	3.8
BUN (6-24 mg/dL)	35	41	47	77	75	40	38	36	34
Serum creatinine (0.50-1.10 mg/dL)	1.70	2.58	4.34	5.71	4.34	3.89	3.40	3.07	2.87
CK (26-192 u/L)		4898	6380	3853	3021	1015	804	676	567
Serum myoglobin (9-83 ng/mL)		>20000	11285	6352	4177	3527	2328	2067	2467
LDH (84-246 u/L)		461							
AST (10-40 u/L)	118	142	416	304	220	136	83	69	60
ALT (12-78 u/L)	90	100	169	152	117	101	69	13	12

## Discussion

Rhabdomyolysis can lead to the development of AKI, often caused by the release of skeletal muscle cell contents into the plasma (myoglobin, aldolase, creatine kinase, aspartate aminotransferase etc.) [6]. CK is used in diagnosing rhabdomyolysis if the serum levels are five to ten times the normal limit [7]. Causes of rhabdomyolysis can be categorized into acquired and inherited causes. Usually acquired causes are commonly seen [2]. The most common acquired causes are substance abuse (34%), medication (11%), trauma (9%), and epileptic seizures (7%) [8]. Very few reports have reported rhabdomyolysis after cardioversion [4-5].

Minor et al. reported a case in which the patient received CPR for almost 90 min, 10 attempts of cardioversion and ultimately requiring hemodialysis with ultrafiltration to manage the acute renal failure. The technetium-99 m pyrophosphate scan was done in the patient as reported by Minor et al. which showed increased area of uptake corresponding with positions of paddles for cardioversion, suggestive of extensive muscle injury in the regions of cardioversion [4] . Hojs et al. [5] reported a patient who suffered MI and received 15 cardioversions. In this report, the patient developed myoglobinuric acute renal failure requiring hemodialysis.

Rhabdomyolysis may induce renal damage due to myoglobin’s direct toxic effect on distal tubular cells or obstruction of the lumen of distal tubules, and results in acute renal failure in up to one-third of the cases [3, 9]. Vanholder et al. reported that in ischemic tissue injury both traumatic and nontraumatic, most of the damage is inflicted after the blood flow into the damaged tissue is restored (reperfusion injury) due to migration of leucocytes into the damaged tissue and production of free radicals [6]. Patients with acute MI were found to have elevated levels of the myoglobin in the urine and serum [10]. Lund et al. did a prospective cohort study to measure serum level of myocardial proteins after cardioversion on 72 patients [11]. The total energy used was 408 +/- 316 J energy. The maximum number of shocks delivered was 4.

The CK rose to more than twice the upper limit of the reference range in 36% of the patients and the peak correlated with the total energy delivered. Peak CK-MB and myoglobin strongly correlated with the total energy delivered and peak energy. Troponin T level did not rise after electric cardioversion but there was minor increase in troponin I [11].

**Figure 1:**
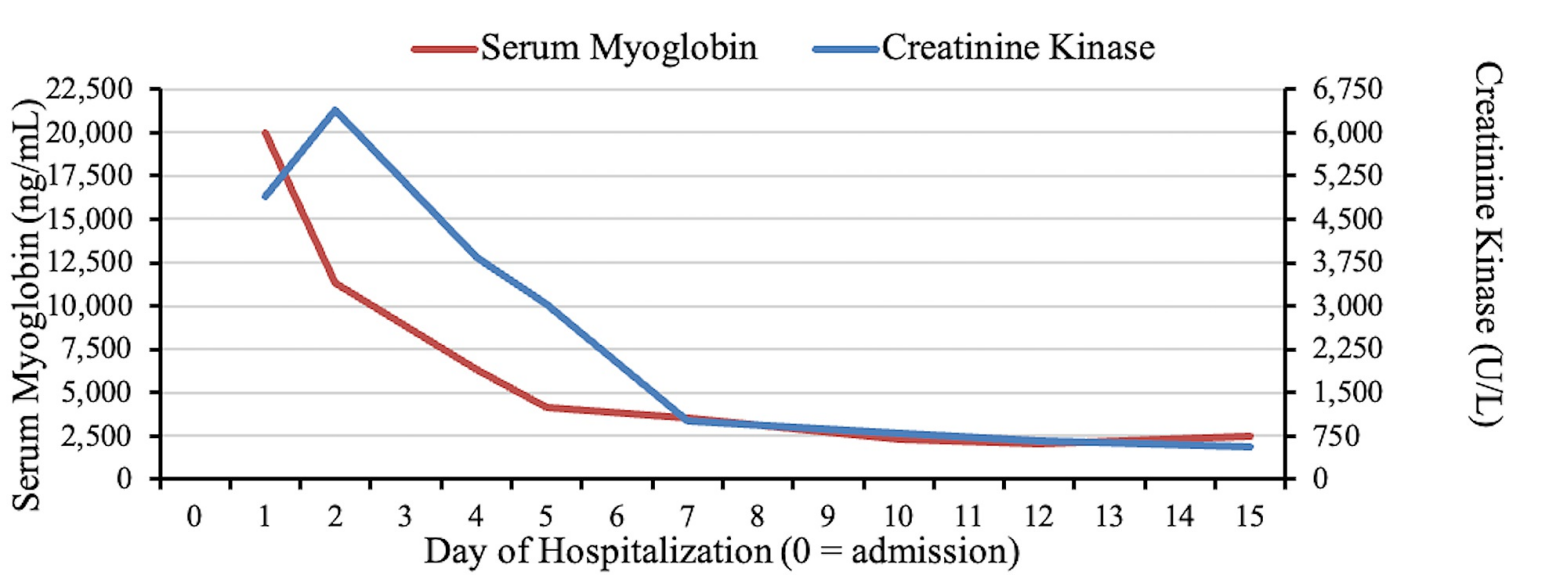
Graphic representation of the level of serum myoglobin and CK over 15 days. CK, creatinine kinase

Another aspect worth mentioning in our case is the high myoglobin level. Studies have shown that serum CK higher than 16,000 U/L are more likely to be associated with renal failure [12-14]. Raju et al. [15] concluded that myoglobin is more higher sensitive test than serum CPK in predicting impending renal failure. The graphic representation of serum myoglobin and CK (Figure 1) demonstrates how myoglobin peaked before CK in our patient. In our case, the CK was elevated but not high enough to definitively say that the patient was progressing towards renal failure. The marked elevation of myoglobin (>20,000 ng/mL) on the other hand was consistent with the findings suggested by Raju et al.’s study, stating that serum myoglobin > 5000 ng/mL is a more sensitive and specific indicator for predicting AKI.

## Conclusions

In our case rhabdomyolysis was confirmed by the marked elevation of myoglobin (>20,000) and more than 10 times the upper limit of CK level. Acute renal failure was confirmed by anuria and increase in serum creatinine. No other cause of rhabdomyolysis was found. There were very few reports and cohort studies reporting MI, CPR, and cardioversion leading to myoglobinuric renal failure. The patient received 1080 J of countershocks leading to myoglobinuric acute renal failure. This case suggests that MI, CPR, and cardioversion can be the cause for rhabdomyolysis and acute renal failure. Several factors might be contributing to the total renal burden of myoglobin in our case including prolonged CPR, repeated cardioversion, and MI. Prompt diagnosis and therapy are essential for adequate management of such patients. In our case, the patient had progressive acute renal failure which was minimally responsive to conservative management requiring dialysis.
